# Validating Efficacy of Shea Nut Oil Extract in Knee Osteoarthritis Patients

**DOI:** 10.1155/2013/147163

**Published:** 2013-12-10

**Authors:** San-Pei Chen, Sui-Foon Lo, Yu-Chia Wang, Tzu-Yi Chou, Kang-Ming Chang, Li-Wei Chou

**Affiliations:** ^1^Department of Physical Therapy and Graduate Institute of Rehabilitation Science, China Medical University, Taichung 40402, Taiwan; ^2^Department of Physical Medicine and Rehabilitation, China Medical University Hospital, Taichung 40447, Taiwan; ^3^School of Chinese Medicine, College of Chinese Medicine, China Medical University, Taichung 40402, Taiwan; ^4^Department of Photonics and Communication Engineering, Asia University, Taichung 41354, Taiwan

## Abstract

*Objectives*. To examine and investigate the efficacy of shea nut oil extract (SheaFlex75) in relation to knee osteoarthritis (OA). *Methods*. Thirty-three patients (age 63.6 ± 5.8 years) with knee OA were recruited. Real-time ultrasound imaging and surface electromyography were used to objectively assess the morphological changes and the activity of vastus medialis oblique (VMO) muscles during a 16-week intervention of SheaFlex75. The intraclass correlation coefficient (ICC) was calculated to examine the reliability of the interscans. A paired-sample *t*-test was used to compare the findings in different stages. The Spearman's rank correlation coefficient was used to examine the relationship between the relevant variables of OA and percentage of thickness change of VMO at different contraction levels. *Results*. The baseline findings showed strong correlation, suggesting that the reliability of interscans at pretest was high. The ability to contract the muscles of the knee to a 30% contraction level showed significant change between the baseline and after 16-week testing, both in terms of morphological changes and muscle activity. Pain scale reported a significant decrease at the 16th week. *Conclusion*. The results suggest that SheaFlex75 can relieve the symptoms of knee OA and can result in improvement of muscle control of the knee.

## 1. Introduction

*Vitellaria paradoxa*, commonly known as the “shea tree,” is a tree of Sapotaceae family, indigenous to Africa. The shea fruit consists of a thin, tart, nutritious pulp, surrounding a relatively large, oil-rich seed, from which “shea butter” is extracted. The butter has been used locally as food, providing a major source of dietary fat. In the West, shea butter is most commonly used in cosmetics [1, 2]. Extracts from the seed have also been used for the treatment of arthritic conditions [3, 4].

Osteoarthritis (OA), also known as degenerative arthritis, degenerative joint disease, and osteoarthrosis, is a group of mechanical abnormalities involving degradation of joints, including articular cartilage and subchondral bone. OA is the most common form of arthritis [5] and the leading cause of chronic disability which affects about twenty-seven million people in the United States [6, 7] and nearly 8 million people in the United Kingdom [7].

It most commonly affects the knee and has an impact on the health-related quality of life of the elderly [5]. Symptoms may include joint pain, tenderness over the inside of knee, stiffness, locking, reduced mobility, atrophy of lower extremities, and decreased walking speed. These functional impairments may reduce a sufferer's general level of exercise and increase the risk of consequent injuries, such as those that might result from a fall.

Patients with knee OA demonstrate different kinematics and kinetics of gait pattern from the healthy adults. These changes may restrict the functional ability of the elderly in daily life, such as walking, stair climbing, navigating obstacles, and standing up. The muscle activation patterns of the key lower extremity muscles involved in the gait were reported to avoid pain and protect the knee from further degeneration. These muscle activities were reported to be different between patients and healthy adults. A study by Stauffer et al. compared patients with knee OA and healthy young adults and reported less knee joint motion, isometric knee strength, and peak ground-reaction force in patients [8]. Suzuki and Takahama [9] and Benedetti et al. [10] observed increased activity of the quadriceps and hamstrings during weight bearing. Kaufman et al. compared the gait patterns during level walking between the patients group (*n* = 139, aged between 30 and 82 years old, grade II OA severity) and healthy controls (mean age = 30 years old) and also found less peak knee motion (54° versus 60°) in the knee OA cohort. The walking speed in the knee OA group was lower than the healthy control [11]. Childs et al. reported that muscle coactivation of the lower extremities showed significant increased and decreased knee excursion during walking [12].

The existing conservative intervention includes medication (such as nonsteroidal anti-inflammatory drugs and steroid joint injections), physical therapy, knee braces, and injections of hyaluronic acid. If disability is significant and the conservative managements are ineffective, surgical knee arthroplasty may be recommended [7]. Recently, another alternative supplement, shea nut oil extract, was proven to be effective. In 1998, US Food and Drug Administration approved shea nut oil as a safe food additive. The traditional Africans have used shea nut oil extracts to treat arthritis, but the mechanism has not been clear. Cheras and his colleagues carried out a 15-week random double-blind OA biomarkers study to compare the effectiveness of SheaFlex70 (a triterpene-rich extract of *Vitellaria paradoxa*) intervention in comparison to placebo groups. The OA biomarkers were found to be significantly decreased in comparison with the placebo group [3]. Experimental and clinical studies showed SheaFlex70 could effectively reduce cytokine, showing improved cartilage retention, bone retention, and pain management [3, 4]. Quadricep strength declines with age, with some evidence to suggest consequent functional impairment. O'Reilly et al. found that patients with symptomatic knee OA demonstrate incomplete activation of the quadriceps and also showed that quadriceps weakness was associated with impaired function. Whether shea nut oil extracts can assist to improve the function of quadriceps was not yet been proven [13].

The few studies that proposed a possible mechanism for the effects of shea nut oil extracts on knee OA have offered comments restricted to the improvement of symptoms or the inflammatory changes within blood. The influence of shea nut oil extracts on patients' functional activity and ability to control or modulate knee function has not yet been explored. This study attempts to do so, investigating the influence of shea nut oil extracts on these functional activities and neuromuscular control of knee OA.

## 2. Materials and Methods

### 2.1. Subjects and Tasks

The group of subjects with OA consisted of volunteers (aged 63.6 ± 5.8 years), 10 males and 23 females, recruited from the outpatient department of the Physical Medicine and Rehabilitation Department, China Medical University Hospital, Taichung, Taiwan. Participants with knee OA had radiographic evidence of bilateral knee OA even if their symptoms were unilateral.

Subjects with knee OA were included if (1) they had been diagnosed with knee OA in line with 1986 American College of Rheumatology clinical criteria, (2) radiographic examination exceeded criteria greater than grade 2 of the Kellgren and Lawrence radiographic criteria (grades ranging from 0 to 4, with 0 being normal and 4 severe OA) [14], (3) they had inner-knee pain, (4) they had morning joint stiffness for over half hour, and (5) they had knee clicking during activities.

Subjects were excluded if they (1) were unable to independently walk or were walking with assistive devices, (2) had a neurological systemic diseases, such as Parkinson's disease, Alzheimer's disease, and Multiple Sclerosis (3) had traumatic injuries or fractures of a lower extremity (hip, knee, or ankle joints), (4) had rheumatoid arthritis or relevant arthritis such as metabolic arthritis (gout), (5) had any surgical intervention on a lower extremity such as arthroplasty, amputation, or ligament reconstruction, (6) had restricted range of motion of lower extremity joints (hip, knee, and ankle), (7) were to undergo physical therapy during the experimental period, or (8) were unable to understand the experimental protocol. All subjects signed an informed consent document approved by the China Medical University Hospital Institutional Review Board prior to taking part in this study.

### 2.2. Experimental Procedure

This is a nonrandomized control, intervention study. Ultrasonography and surface electromyography (s-EMG) were used in this study to provide the objective assessment of the changes to muscle and to investigate the influence of treatment with shea nut oil extract (SheaFlex75). Each participant was requested to attend three sessions to establish baseline conditions and then for assessment after 8 weeks and after 16 weeks. The intervention protocol was to take 6 pills per day for 16 weeks. The morphological changes of muscles around the knees and the ability to control muscles in different tasks were examined at each of the three sessions. The subjective findings were assessed by subjective pain intensity and modified Lequesne index to assess the pain and functional impairment. To avoid intraexaminer difference, the same examiner assessed the questionnaires and real-time ultrasound applications. The other two examiners performed the EMG applications and instruction.

### 2.3. Subjective Pain Intensity, Visual Analog Scales (VAS)

Participants were requested to scale the subjective intensity of pain using the VAS. VAS is an assessment tool for patients to self-assess pain intensity. This tool is widely applied clinically to assess the improvement of pain in patients with musculoskeletal dysfunction. The questionnaire was composed of a 10 cm continuous line between two end-points. One end is 0 and the other end is 10. The “0” indicates no pain and the “10” indicates the most severe pain (intolerable pain). Each patient specifies his/her level of agreement to a statement by indicating a location along this line.

### 2.4. Modified Lequesne Index in Knee Osteoarthritis

The Modified Lequesne index in knee OA, which was developed in France in 1970 and was published in 1980, is an assessment tool for the functional characteristics of knee OA. Eleven questions are used to evaluate these characteristics, including knee pain, stiffness, walking, squatting, and stair climbing. The sores are between 0 and 24. Faucher et al. reported high reliability and validity of the questionnaire in the context of determining symptom changes and function assessment of knee OA patients [15].

### 2.5. Rehabilitative Ultrasound Imaging (RUSI)

Physicians use ultrasound as a diagnostic imaging modality and also as “interventional ultrasound” or “invasive ultrasound” to assist and guide procedures during examination or surgery [16]. For the rehabilitation professional, ultrasonography is not a stand-alone assessment or treatment tool but is incorporated alongside existing clinical skills. In such a context, it is primarily deployed for viewing both static and dynamic muscle performance to investigate neuromuscular control and identify underlying dysfunction. These findings provide foundation information for assessment and treatment of neuromuscular control and the design of training regimes. As used by therapists, it is termed “rehabilitative ultrasound imaging” [17]. In general, RUSI provides a relatively low-cost and noninvasive way to examine the muscles, but its sensitivity for effective detection of muscle changes as an outcome measurement in an intervention study remains to be determined.

**Figure 1:**
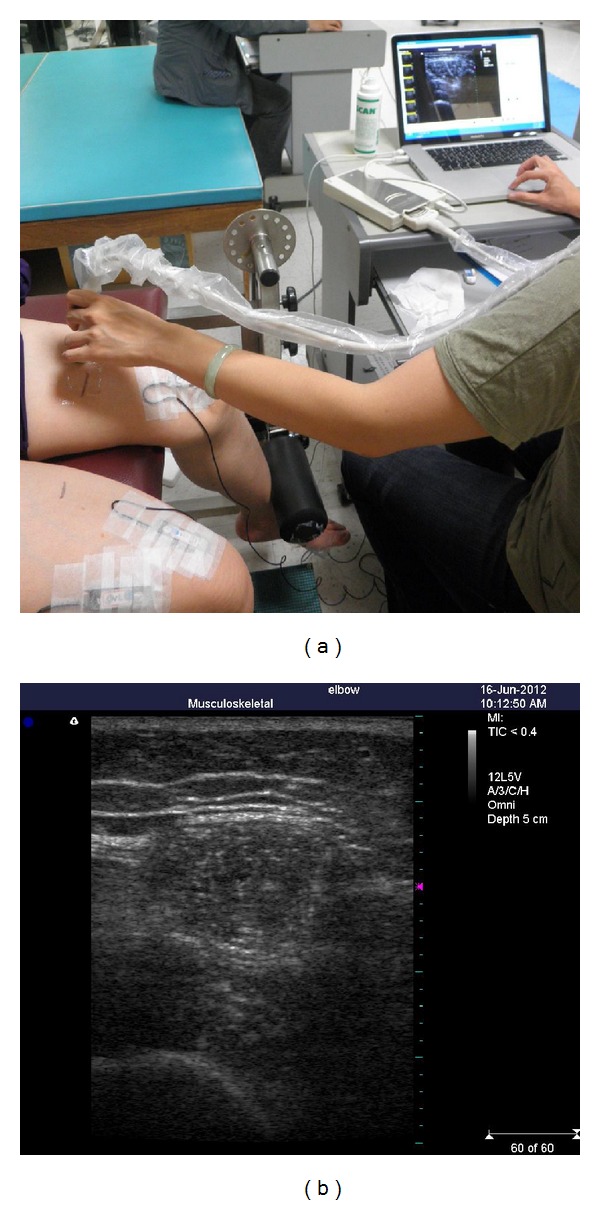
The examiner used the linear transducer to capture the image of the vastus medial (VM) at each muscle status (a) and the scanned image (b).

**Figure 2:**
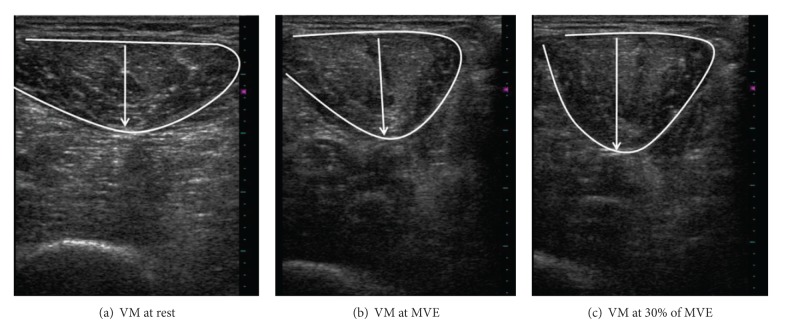
The scanned images of the vastus medial (VM) at three different muscle statuses. The overlay depicts the shape of VM and the distance between opposite points measured as the thickness of the VM.

In this study, ultrasound images were taken using a linear transducer (>7.5 MHz). Images of the vastus medial (VM) were captured both at rest and in contracted states at two different knee positions (see Figure 1). All images were downloaded to a computer to be measured offline using Image “J software 1.4.” During scanning of the knees, each participant was positioned in an erect sitting position, on a NK table using a device to standardize the position of knee. The site for scanning was at the distal height at one-third of the distance between anterior superior iliac spine and medial tibial plateau, in a standing posture. The asymptomatic or less symptomatic side was examined first, and each task was performed three times. Rest images were taken bilaterally at the position of knee flexion at 90 degrees. Subsequently, subjects were requested to activate knee extensors to the maximum by extending the knee against the pad at the same posture. The examiner then adjusted the torque arm of NK table to 60 degrees. The maximal voluntary effort (MVE) and 30% of MVE were performed at this position, separately. The same protocol was replicated for the other leg. As expected, muscle thickness increased with the level of effort. Since the actual thickness of muscle was different (see Figure 2), percentage changes were useful for comparing the general changes within the three sections. The equation is

(1) (Thickness  at  contraction  status−Thickness  at  rest)Thickness  at  rest  ×100%.

### 2.6. Surface EMG Protocol

**Figure 3:**
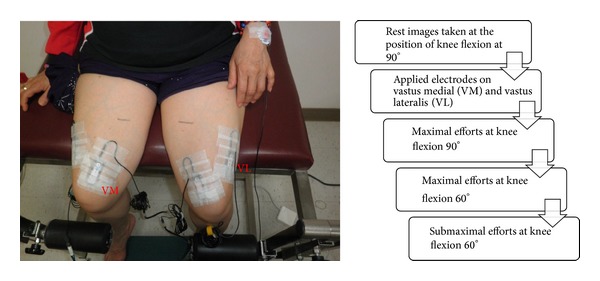
Examples of electrode positions for VM and VL placement. The ultrasound and s-EMG data is synchronously collected. The experimental protocol is listed, right.

The EMG signals from 8 muscles of the bilateral vastus medial (VM), vastus lateralis (VL), medial hamstring (MH), and gastrocnemius (GM) were collected by the band-pass filter at 2–400 Hz and at a sample rate 1000 Hz (using Biopac Systems MP150 and EMG 100B, Biopac, USA). Following the SENIAM skin preparation protocols, the alcohol swabs were used to wipe skin and then the electrodes were placed. The electrodes were oriented on the centre of the muscle belly in a longitudinal fashion in the direction of the muscle fibers. Placement of the electrode was facilitated by palpating the muscle as the subject contracted the muscle against resistance (Figure 3). A reference electrode was placed over the radius styloid process of the right wrist. Reusable electrodes were used to detect the signals from muscles.

The electrodes over the VM and VL were placed with the subject in a 90-90 sitting posture on the NK table, and then the MVIC of these two muscles collected with ultrasound synchronously (Figure 3). Each participant was requested to extend the tested leg against the pad of N-K table continuously for 5 seconds. Data for the MVIC and 30% MVIC of VM and VL were collected at 60 degrees of knees separately. The electrodes over the right and left hamstrings were placed with the subject in a prone position. Each participant was requested to flex the knee maximally against resistance from the examiner. The magnitudes of muscle forces were smoothed using a moving root-mean-square (RMS) filter with a 25 ms window. To facilitate comparison among sessions, the s-EMG was normalized to the mean of the RMS values obtained during three 3-seconds maximal voluntary isometric contractions of each of the muscles [18].

### 2.7. Data Analysis Strategy

The age, body mass, height, and body mass index (BMI) of the subjects are presented in the descriptive statistics. The analysis of ultrasound imaging data was conducted using the statistical methods shown below, and the descriptive findings of the measurements taken are then given. The intraclass correlation coefficient (ICC) was considered to be an appropriate statistical method for analysis of repeated measurements of muscle thicknesses [19–21] and was calculated to examine the reliability of the inter-scans. The Spearman's rank correlation coefficient was used to examine the relations among the variables of OA severity level, pain duration, pain scale, and percentage change in thickness of VMO at different states of contraction. A paired-sample *t*-test was used to assess the difference between the percentage change in thickness and muscle amplitude. A *P* value of less than 0.05 was taken to indicate significant difference.

## 3. Results

### 3.1. Descriptive Findings

In this study, 33 patients completed the three sections of the experimental protocol. The demographic data of the participants are listed in Table 1. The descriptive findings of the percentage change in thickness among rest and various contraction states of the VM are listed in Table 2.

### 3.2. Reliability of RUSI in OA Patients

Inter-scan reliability at the baseline secession was reported as moderate (ICC > 0.85) to approve the stability of skills, indicating acceptable levels of consistency of subject behavior during the trials. The Spearman's rank correlation coefficient was used to assess the correlations and the presence of other possible variables which may have been affecting the muscle contractions. The OA severity level, pain duration, and pain scale (*P* > 0.05) showed no significant difference with the percentage of muscle contractions. The pain duration was defined in three groups: group 1 as 6–12 months, group 2 as 1–5 years, and group 3 as more than 5 years (Table 3).

### 3.3. Comparisons of the Findings within the Three Trials

**Figure 4:**
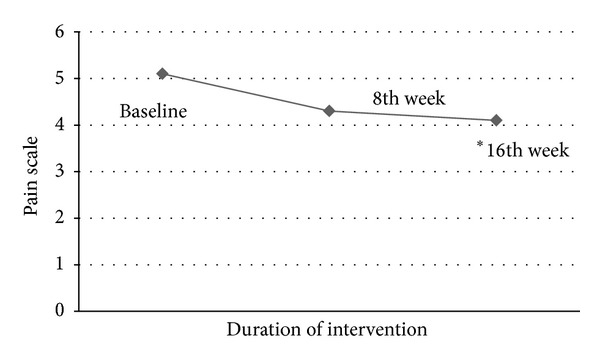
Symptom Change of VAS during the three trials (∗ indicates significant difference in comparison with the baseline).

The paired-sample *t*-test was used to compare the findings at baseline, after 8 weeks, and before 16 weeks. Significant findings in relation to the percentage change in thickness at submaximal efforts between the baseline and after 16 weeks were reported (*P* = 0.02) (Table 4). The modified Lequesne index includes 4 sections; pain, stiffness, mobility, and total work. The paired-sample *t*-test was used to compare values acquired from subjects during the three sessions. The pain section and stiffness section showed a significant difference, with *P* = 0.01 and *P* = 0.04, respectively (Table 4). The VAS pain was reported as significantly different (*P* = 0.03) between the baseline and after 16 weeks sessions, showing declined signs as the intervention progressed (Figure 4). The average amplitude of VM and thickness change of VM were compared among the three sessions as well, and significant findings were reported between the baseline and after 16-week stages (*P* = 0.04) (Table 4).

## 4. Discussion

### 4.1. Summary of Important Findings in This Study

The symptom relief and improved muscle control were observed both in ultrasound and s-EMG findings at sub-maximal effort after the 16-week intervention. This indicates that shea nut oil extract can significantly relieve the symptoms in question and the subject's response to muscle activity and further the morphological changes after 16-week intervention.

### 4.2. The Relation between Muscle Thickness and Muscle Activity

Changes in muscle thickness from the resting state to a contracted state are considered to reflect changes in muscle activity level, although the linearity of the relationship is controversial [22, 23]. Although muscle thickness change and amplitude of EMG are likely to be a positively correlated, the relationship is certainly not simple enough to be linear and perhaps only marginally curvilinear over a small range. As such, the relationship between muscle thickness and EMG remains inconclusive [24–26]. Strasser and his colleagues [27] carried out a randomized and observer blind study to investigate whether the quadriceps measured by real-time ultrasound correlated with isometric maximum voluntary contraction force (MVC) of quadriceps in young and old sarcopenia. They found that thickness of VM had the best correlation with MVC in the elderly and showed that measurement of muscle thickness, especially of VM by real-time ultrasound, is a reliable method for monitoring the extension of quadriceps. These clinical data support our finding that thickness measurement by real-time ultrasound could be a reliable and valid technique as an objective assessment to monitor muscle function. As the significant morphological changes and muscle amplitudes only occurred at the sub-maximal effort, it can be suggested that the ability to voluntarily control or adjust the strength has been improved, while no claim relating to increased capability at maximal effort can be made.

### 4.3. The Possible Analgesic Effect of the Shea Nut Oil Extract

Postexercise induced muscle soreness (PEMS) is a dull, aching pain combined with tenderness and stiffness [28], usually following unaccustomed eccentric exercise [29, 30]. Maclntyre and his colleagues found that neutrophil levels were greater in the exercised muscle than in nonexercised muscle and the delayed onset muscle soreness was increased from 0 to 48 hours, and eccentric torque decreased from 2 to 24 hours. Significant relationships were found between interleukin 6 (IL-6) levels at 2 hours and delayed onset muscle soreness at 24 hours after exercise. These findings suggest a relationship between damaged to the contractile proteins and inflammation. A significant relationship of the severity of PEMS after eccentric exercise and IL-6 has been shown. Intense eccentric exercise results in high tension and mechanical damage in the muscles that is followed by increased amounts of IL-6 release, which corresponds to the time-course development of muscle soreness [31]. Arendt-Nielsen and his colleagues [32] conducted a randomized, prospective, double blind, placebo-controlled, parallel group study demonstrating that prophylactic supplementation by shea nut oil extract significantly relieves muscle tenderness associated with intensive eccentric exercise, most likely via reduction of proinflammatory cytokine IL-6.

In the present study, clinical data showed that the knee OA symptoms, in particular pain and stiffness, improved significantly after the 16-week intervention. The preliminary results have proven an analgesic effect of the shea nut oil extract that is consistent with the currently existing studies [3, 4]. Pain reduces strength and endurance of the muscles by inhibiting muscle recruitment and thus impeding motor control [33, 34]. Muscle contraction should be improved and patients may regain part of their motor control ability.

### 4.4. The Possible Biologic Mechanisms of the Shea Nut Oil Extract in Knee OA

The study by Alander and Andersson [35] concluded that fractionated shea butter caused a significant reduction of the inflammatory response of human keratinocytes in comparison to croton oil. The active agent of shea tree extracts contains triterpenes, derived from the seed of the shea tree, *Vitellaria paradoxa*. The most abundant triterpenes are butyrospermol, lupeol, and the *α* and *β*-amyrin, in addition to their dihydroderivatives. Findings that the triterpenes have an anti-inflammatory effect and can suppress NF-*κ*B activation were noted in previous studies [36, 37].

Cheras et al. conducted a randomized double-blind placebo-controlled trial to investigate the potential modes of action of the triterpene-rich shea tree nut extract, in the treatment of osteoarthritis. They concluded that treatment with shea nut oil extract during the 15-week duration of the study showed that subjects with elevated biomarkers presented a range of anti-inflammatory and chondroprotective effects together with a potential for beneficial modulation of bone formation. Significant reductions in inflammation were shown by decreased TNF-alpha, hsCRP, and IL-6, which all fell by over 20% in the treatment group and the reduction in C-telopeptide fragments of type II collagen (CTX-II) over the 15-week study across the entire active group and in the active subgroup with high levels at baseline. This is consistent with chondroprotective activity of shea nut oil extract [3]. Their findings that inflammation was decreased over 15 weeks are consistent with the current results that the symptoms and muscle functions are improved significantly after a 16-week intervention.

### 4.5. The Possible Influences of SheaFlex75 on Knee OA

Hodges and Moseley reviewed and summarized a number of mechanisms that have been proposed to explain the effect of pain on motor control in the spine and extremities. The majority of available hypotheses is broadly consistent with two main theories: the pain-spasm-pain model and the pain adaption model. The pain-spasm-pain model was suggested to be too simplistic, and so the current experimental and clinical data offer support for the pain adaption model [33]. Another review by Sterling et al. reported that clinical and basic science investigations have provided evidence in relation to changes of motor function under the effects of pain, including both increases and decreases in muscle activity, along with alterations in neuronal control mechanisms, proprioception, and local muscle morphology. A new model (the neuromuscular activation model) was used to explain how the patterns of muscle activation and recruitment are altered in the presence of both acute and chronic pain. The alterations seem to particularly affect the ability of muscles to perform synergistic functions related to maintaining joint stability and control [34]. These discussions address the notion that pain causes motor control changes, ranging from changes in recruitment to reduced strength and endurance of the muscles, and that the possible patterns are hyperactivity and hypoactivity.

Cheras et al. found that shea nut oil extract treatment over the 15 weeks of their study was effective in decreasing inflammation responses and relevant symptoms [3]. Knee OA is a chronic disease, and muscle function and bony structure progressively deteriorate over time, altering muscle activation and strength of key lower extremity muscles during weight-bearing activity. Quadricep muscle strengthening is a common management of knee OA in that it can delay OA progression. The ability to control and adjust the function of the quadriceps muscles may thus assist the treatment of knee OA. According to existing studies, the thickness change in real-time ultrasound and s-EMG activity of the back and VM muscles correlates linearly, showing that the greater the thickness change, the greater the amplitude of muscle activation [22–27].

In this study, ultrasonography is used primarily for viewing both static and dynamic muscle performance, to define neuromuscular control of normal muscles and those that present specific alteration caused by underlying dysfunction. The effectiveness of shea nut oil extract on the morphological changes of muscles in images is not clear. Herein, the present findings indicate that the contractile function of the knee extensors during sub-maximal effort was significantly changed after 16-week stage of intervention, compared with the baseline finding, but no significant difference was found between the baseline and after 8-week intervention. Maximum effort of the knee muscles was not applied during functional activities, and sub-maximal effort was used to control and coordinate the agonist and antagonist muscles to achieve the goal.

How changes in control relate to the fear associated with pain is still in question. It is proposed that fear of causing pain is critical in behavioral and motor output, and the fear-avoidance model is gaining considerable support in the literature [34]. The fear avoidance model argues that fear of pain and (re)injury prevents the normal return to activity, leading to deconditioning and disability, as reported by patients with lower back pain [33]. Patients with OA may behave similarly. If so, it seems reasonable that if deconditioned strength and endurance may not be regained by simple pain alleviation, they may be obtained through progressive engagement with exercise. As for the subjective functional assessment, patients' mobility and total symptoms were not reported as significantly improved. The findings indicated that improvement in symptoms did not immediately manifest in the activities of daily living, perhaps due to habitual compensative strategies. Our studies found that the ability to control and coordinate the contractile components was improved in the sub-maximal effort of muscles, but not in the maximal voluntary effort. It can be concluded that SheaFlex75 can alleviate pain and improve the contractile ability of knee muscles in knee OA after 16-week intervention.

### 4.6. The Limitation of This Study

Inevitably, difficulties were encountered during the study. It was designed to be a case-control intervention to investigate the validity of a proposed effect of shea nut oil extract on knee OA. Due to the difficulties in the recruitment of patients and commitment to the 16-week intervention, the study design did not include a placebo group. In addition, knee OA is a chronic and progressive disease; it was recognized that some patients may drop out for personal reasons or because they wish to accept another medical intervention such as physiotherapy or acupuncture to relieve their symptoms.

The morphological changes and muscle activity of VMO was used as the main outcome measurement. However, it is recognized that the possibility that of muscle imbalance may influence the ratio of the change in thickness and amplitude change in the quadriceps. The VL data was not collected as completely as that for the VM, although Strasser et al. reported a strong correlation between the strength and thickness of the VM in old sarcopenia [27]. That these findings can be applied to and properly support the notion of this study is not proven, due to the different experimental protocol and population.

A further complicating feature of the trials was that patients were observed to use compensative strategies to achieve the task set. It is suggested that this could be the result of fear of resulting pain or of habitual strategies and may have influenced observed muscle control.

As for the issue of thickness measurement, the longitudinal distance between muscle borders of the VM was calculated at the rest and during contraction. However, as the VM is an oval-shaped muscle in the transversal view, the ratio of cross-section area of the VM may be a more precise measurement. The individual muscle sizes varied, and some images were not within the range of the scans. Therefore, the measurement of longitudinal distance of VMO was made. These limitations and practical issues may provide opportunities for future improvement.

## 5. Conclusions

The effectiveness of treatment of knee OA using shea nut oil, an extract from the indigenous African *Vitellaria paradoxa* tree, is proven. After sufficient dosage and intervention, its effects include decreased inflammation, increased collagen, amelioration of pain, and improved muscle function. Although improved muscle function was observed, including greater control and an increase in muscle strength to achieve a functional goal, the subjective feeling of improvement in the activities of daily living was not significant.

The findings have proven the efficacy of shea nut oil extract as a complementary option to improve the symptoms and function in relation to knee OA.

## Figures and Tables

**Table 1:** Demographic data of participants completing the three studies (*n* = 33).

	Mean	SD
Age (y/o)	63.6	5.8
BW (kg)	66.8	11.8
Height (m)	1.6	0.1
BMI (kg/m^2^)	26.5	4.5

**Table 2:** Descriptive findings of the percentage change in thickness among rest and different contraction statuses in VM.

	Percentage change in thickness	
	Mean (%)	SD (%)
Rt_90_dif	19.5	14
Lt_90_dif	17.5	12.9
Rt_60_dif	16.5	11.8
Lt_60_dif	18.3	13.5
Rt_60_sub_dif	15.5	12.3
Lt_60_sub_dif	15.6	12.6

Rt: right; Lt: left; 90: MVC at knee flexion 90°; 60: MVC at knee flexion 60°; submax: submaximal effort (30% of MVC) at knee flexion 60°.

**Table 3:** The correlation between muscle thickness change and other possible contributary factors.

Baseline data	Correlation coefficient	*P* value
OA severity level		
PTC at 90	−0.2	0.15
PTC at 60	−0.09	0.51
PTC at submax	−0.02	0.88
Pain duration		
PTC at 90	0.03	0.85
PTC at 60	−0.11	0.42
PTC at submax	−0.15	0.26
Pain scale		
PTC at 90	0.06	0.69
PTC at 60	0.05	0.73
PTC at submax	0.05	0.74

PTC: percentage of thickness change; 90: MVC at knee flexion 90°; 60: MVC at knee flexion 60°; submax: submaximal effort (30% of MVC) at knee flexion 60°.

**Table 4:** Comparison of the findings at baseline, after 8 weeks and after 16 weeks.

Pair *t*-test	Baseline—after 8 weeks (*P* value)	Baseline—after 16 weeks (*P* value)
RUSI		
90	0.11	0.81
60	0.34	0.97
60 submax	0.73	0.02*
EMG		
90	0.24	0.12
60	0.6	0.32
60 submax	0.08	0.04*
Questionnaires		
Pain	0.03*	0.01*
Stiffness	0.07	0.04*
Mobility	0.17	0.08
Total work	0.57	0.25

*P < 0.05, significance.

90: MVC at knee flexion 90°; 60: MVC at knee flexion 60°; submax: submaximal effort (30% of MVC) at knee flexion 60°.
